# Foot and ankle biomechanics play a role in biomechanical response to lateral wedge insoles

**DOI:** 10.1186/1757-1146-7-S1-A39

**Published:** 2014-04-08

**Authors:** Richard K Jones, Graham J Chapman, Matthew J Parkes, Laura Forsythe, David T Felson

**Affiliations:** 1 Centre for Health Science Research, University of Salford, Greater Manchester, M6 6PU, UK; 2 Institute of Rheumatic and Musculoskeletal Disease, University of Leeds, Leeds, UK; 3 Arthritis Research UK Epidemiology Unit, University of Manchester, Manchester, UK

## Background

Lateral wedge insoles have consistently shown to reduce the external knee adduction moment (EKAM) in medial knee osteoarthritis (OA) patients; although there is evidence that certain patients have a paradoxical increase in EKAM. This may be a key factor in determining clinical response and thus identifying and understanding why these patients increase EKAM is critical for prescribing the correct treatment for these patients. Previous evidence has suggested that foot and ankle biomechanics play a role in reducing EKAM by shifting the centre of foot pressure (COFP) laterally and increasing the valgus orientation of the calcaneus, which shortens the lever arm in respect of the knee, thus reducing the EKAM. To date, patients have been studied irrespective of biomechanical response to lateral wedge insoles. In this study we investigated whether dynamic ankle biomechanics can assist in identifying and explaining why some patients increase EKAM and other decrease EKAM when wearing a lateral wedge.

## Methods

Participants diagnosed with medial knee OA were recruited to the study. Each participant underwent a 3D kinematic (Qualysis OQUS, Gothenburg, Sweden) and kinetic (AMTI, USA) analysis whilst walking in a control shoe and the two different lateral wedge insoles which were inserted bilaterally into the control shoe. The order of testing was randomised. We classified participants as biomechanical responders (responder) if participants decreased EKAM under *both* lateral wedge conditions compared to the control shoe. We defined biomechanical non-responders (non-responder) as those whose EKAM increased when wearing *both* lateral wedges compared to the control shoe. Fixed-effects multiple linear regressions were used to test for effects of the lateral wedge on coronal ankle variables, in both wedge conditions and subsequently when dichotomising individuals into biomechanical responder and non-responders. Finally, logistic regression was performed to see which coronal ankle variables, measured in the control condition only, could predict response to EKAM.

## Results

Of the 70 participants studied (43 male), 20% increased their EKAM and 54% decreased their EKAM. Both pairs of lateral wedge insoles caused the foot to be in a significantly more everted position compared to the control condition with one insole greater. Change in ankle angle excursion significantly predicted EKAM change with lateral wedge insoles. Additionally, individuals with a higher peak ankle eversion angle (OR 1.31; 95% CI 1.019 to 1.703; *p* = 0.036) or a higher eversion angle at peak EKAM (OR 1.31; 95% CI 1.02 to 1.70; *p* = 0.037) during the control condition were more likely to classified as a biomechanical responder to the lateral wedges.

## Conclusions

In conclusion, we have demonstrated for the first time that coronal plane foot and ankle biomechanical measures are key mechanisms for the reduction of EKAM when wearing lateral wedge insoles. Furthermore, our findings also demonstrate that coronal plane ankle biomechanical measures under the control condition predict if an individual is likely to decrease their EKAM when wearing lateral wedge insoles. These findings may provide future insights into determining who will respond to lateral wedge insoles.

## Trial registration

ISRCTN: 83706683.

